# Machine Learning-Based Risk Prediction of Critical Care Unit Admission for Advanced Stage High Grade Serous Ovarian Cancer Patients Undergoing Cytoreductive Surgery: The Leeds-Natal Score

**DOI:** 10.3390/jcm11010087

**Published:** 2021-12-24

**Authors:** Alexandros Laios, Raissa Vanessa De Oliveira Silva, Daniel Lucas Dantas De Freitas, Yong Sheng Tan, Gwendolyn Saalmink, Albina Zubayraeva, Racheal Johnson, Angelika Kaufmann, Mohammed Otify, Richard Hutson, Amudha Thangavelu, Tim Broadhead, David Nugent, Georgios Theophilou, Kassio Michell Gomes de Lima, Diederick De Jong

**Affiliations:** 1 Department of Gynaecologic Oncology, St James’s University Hospital, Leeds LS9 7TF, UK; yong.tan@nhs.net (Y.S.T.); gwendolyn.saalmink@nhs.net (G.S.); albinazubayraeva@gmail.com (A.Z.); racheal.johnson2@nhs.net (R.J.); angelika.kaufmann@lthtr.nhs.uk (A.K.); motify@nhs.net (M.O.); richard.hutson@nhs.net (R.H.); amudha.thangavelu@nhs.net (A.T.); tim.broadhead@nhs.net (T.B.); david.nugent@nhs.net (D.N.); georgios.theophilou@nhs.net (G.T.); diederick.dejong@nhs.net (D.D.J.); 2 Department of Chemistry, Federal University of Rio Grande do Norte, Natal CEP 59078-970, Brazil; raissavanessa781@hotmail.com (R.V.D.O.S.); daniellucas77@hotmail.com (D.L.D.D.F.); kassiolima@gmail.com (K.M.G.d.L.)

**Keywords:** Machine Learning, surgical cytoreduction, Critical Care Unit, ovarian cancer, Graphical User Interface

## Abstract

Achieving complete surgical cytoreduction in advanced stage high grade serous ovarian cancer (HGSOC) patients warrants an availability of Critical Care Unit (CCU) beds. Machine Learning (ML) could be helpful in monitoring CCU admissions to improve standards of care. We aimed to improve the accuracy of predicting CCU admission in HGSOC patients by ML algorithms and developed an ML-based predictive score. A cohort of 291 advanced stage HGSOC patients with fully curated data was selected. Several linear and non-linear distances, and quadratic discriminant ML methods, were employed to derive prediction information for CCU admission. When all the variables were included in the model, the prediction accuracies were higher for linear discriminant (0.90) and quadratic discriminant (0.93) methods compared with conventional logistic regression (0.84). Feature selection identified pre-treatment albumin, surgical complexity score, estimated blood loss, operative time, and bowel resection with stoma as the most significant prediction features. The real-time prediction accuracy of the Graphical User Interface CCU calculator reached 95%. Limited, potentially modifiable, mostly intra-operative factors contributing to CCU admission were identified and suggest areas for targeted interventions. The accurate quantification of CCU admission patterns is critical information when counseling patients about peri-operative risks related to their cytoreductive surgery.

## 1. Introduction

Cancer of the fallopian tube, ovary, or peritoneum (EOC) is the leading cause of death from gynaecological malignancy in the western world [1]. Over 70% of women diagnosed with EOC have advanced stage disease at presentation (International Federation Obstetrics and Gynaecology FIGO stage 3–4) [1]. The gynaecological oncology community is now recognizing high grade serous ovarian cancer (HGSOC), as yet the most prevalent, as a single clinical entity. For such an aggressive subtype, identification of novel, ideal biomarkers that strongly correlates with the stage and may be effective for early diagnosis, remains critical [2]. The cornerstone of treatment includes a combination of surgical cytoreduction and platinum-based chemotherapy aiming at elimination of all visible disease, ideally reaching a total macroscopic tumor clearance. When the cancer is at an advanced stage, the debulking surgery often requires prolonged surgical times and possible multi-visceral resections, necessitating Critical Care Unit (CCU) support, and prolonged hospitalization [3]. Risk-prediction models of severe post-operative complications after ovarian cytoreductive surgery have been thoroughly proposed [4].

Routine post-operative CCU admission has been considered a standard of care for such patients at risk of increased peri-operative morbidity and mortality, and post-operative complications [5]. Patient-related pre-operative risk factors associated with significant peri-operative outcomes have been comprehensively studied [6]. There is currently a growing interest in monitoring CCU admissions, and performance has been compared across different trusts to improve the standards of care. In the realm of precision medicine, innovative data mining technologies, such as Machine Learning (ML), can be clearly used for monitoring quality improvement and effectiveness of delivery of modern ovarian cancer care. The ML approach for outcome prediction based on fully curated clinical data has been applied in a variety of hospital settings [7]. We previously developed ML algorithms to improve the prediction accuracy of complete cytoreduction in advanced stage HGSOC patients [8]. In addition, we highlighted the importance of feature selection for the ML-based two-year prognosis estimation in the same population [9]. The usefulness of ML as a prognostic toll in the ovarian cancer environment has been increasingly demonstrated [10]. 

Accurate prediction is difficult with conventional statistics, because patient characteristics show a multidimensional and non-linear relationship. Machine Learning methods could be helpful in monitoring CCU admissions to improve standards of care. In an era of stringent economics, CCU admission is often arbitrary, driven by local practice or bed availability. It remains heavily at the surgeon’s discretion, as post-operatively, the gynaecologic oncologist is mainly responsible for daily monitoring and decision making with regards to the patient’s recovery [5]. International comparison studies have suggested that patterns of CCU admission after surgery are inconsistent, and the value of such admissions routinely may be nuanced [6]. While focusing on the cost-effectiveness of CCU utilization after major surgeries, timely identification of those patients, who will benefit from CCU support, remains a major challenge [11]. Lately, the introduction of maximal surgical effort to achieve complete cytoreduction without compromising peri-operative management and subsequent recovery requires the availability of CCU beds [12]. This paradigm shift has prompted the development of tools to accurately predict CCU admission following cytoreductive surgery. To inform consent and enhance decision making, we developed and validated an ML-based risk score to predict CCU admission of advanced stage HGSOC patients undergoing surgical cytoredution. We hypothesized that CCU admission of HGSOC patients is multifactorial and could be accurately predicted by using ML algorithms. The primary outcome was the prediction accuracy of several ML prediction methods, based on a set of performance metrics, and the performance comparison with the conventional logistic regression (LR) analysis. The secondary objective was factor refinement for CCU admission and exploration of the associations between CCU admission, severe post-operative complications, hospital stay, and 30-day hospital re-admission. 

## 2. Materials and Methods

Prospectively registered data in the hospital-wide Patient Pathway Manager (PPM) database from 291 consecutive HGSOC women, who underwent cytoreductive surgery at St James’s University Hospital, Leeds by a certified Gynaecologic Oncology Surgeon from January 2014 to December 2019 was analyzed. This database was developed internally for clinical trials and integrated with an electronic patient record system [13]. Our hospital is a tertiary centre, recently accredited by the European Society of Gynaecologic Oncology (ESGO) as a centre of excellence for ovarian cancer surgery. The staging was reported by the 2014 International Federation of Gynaecology and Obstetrics classification [14].

Women underwent either primary debulking surgery (PDS) or 3–4 cycles of neoadjuvant chemotherapy (NACT) followed by interval debulking surgery (IDS) if they had: FIGO stage 4 disease; poor performance status (PS); uncertainty about the possibility of optimal tumor removal. Only HGSOC patients with at least one pre-treatment CA125 measurement were included in the study. Patients aged < 18 years, as well as those with progressive disease, synchronous primary malignancy or secondary cytoreduction, were excluded. Post-operative CCU admission was considered for high-risk HGSOC patients, who were scheduled to undergo complex major surgery, including multi-visceral resections or undertook pre-operative cardiopulmonary exercise (CPEX) fitness testing, if they were deemed high risk at pre-assessment [15].

### 2.1. Candidate Variables for Prediction Model

Candidate predictors were selected a priori from two domains:

- Patient: age, year of diagnosis, body mass index (BMI), pre-operative co-morbidities (Charlson Co-morbidity Index (CCI)), Eastern Co-operative Oncology Group (ECOG) performance status (PS), and pre-operative albumin.
- Operative factors: timing of surgery (PDS or IDS), operative time (OT), surgical complexity score (SCS), bowel resection with stoma formation (yes/no), residual disease (RD), and estimated blood loss (EBL).

All these variables are widely available at tertiary centres. Based on clinical experience, they have been previously shown to be independent predictors of post-operative morbidity and mortality for ovarian cancer patients [4]. Severe post-operative complications were reported according to the Clavien-Dindo morbidity score (grade 3–5) [16]. Only several surgical site infections (SSIs) were recorded. The CCI was categorized as: 0, 1, 2 with higher scores indicating greater co-morbidity [17]. The SCS was assigned based on the Aletti classification as low, intermediate, and high [18]. Outcomes also included rate of CCU admission, length of hospital stays, and hospital re-admission, defined as admission to any hospital for longer than 24 h within 30 days of discharge.

Descriptive statistics were displayed by frequency and percentages for binary and categorical variables and by means and standard deviation s(SD) or medians (with lower or upper quartiles for continuous variables). The Chi-square test was performed for categorical variables. The Fischer’s exact tests were used for binary variables. All analyses were performed with IBM^®^ SPSS^®^ 26.0 Statistics Software for Windows (IBM, New York, NY, USA).

### 2.2. Feature Reduction and Selection

Our objective was to use all the available data to maximize the generalizability of our results. Feature selection from the candidate predictors aimed to optimize the predictor group by finding the smallest independent set of variables with the greatest association to the outcome in question, in order to ensure best performance and to minimize over-fitting. We used standard feature selection strategies, including dropping-O variance and highly correlated variables. All clinical variables were evaluated individually, one by one, with the vector of CCU classification responses. Forward selection and backward stepwise regression were then employed to select independent variables for inclusion in the final models. Variables with a threshold *p*-value < 0.05 and 95% Confidence Intervals (CI) were considered to be statistically significant and were included in the predictive model. To re-value two variables as more relevant, and to optimize the latent collinearity and avert variable over-fitting, a correlation analysis was evaluated by assessing the determination coefficient (R^2^) and the root mean square error of cross validation (RMSECV). A poor correlation between variables was observed if the R^2^ was low or the RMSECV was elevated. Variables with a threshold of >0.6 were eliminated to determine the final input variables for the model. Highly inter-correlated variables were grouped, and variables with no mutual optimization were dropped. The resulting rankings were used to select the set of features that led to the highest prediction accuracy. Subsequently, the optimal number of important features resulting in the highest prediction accuracy was identified. For feature importance, we employed resources for assigning weight to system variables. That is, for any chosen ML method, we assigned different weights to different variables. 

### 2.3. Model Development and Training

The model development followed a standard data science approach. The raw clinical data were imported into MATLAB environment (MathWorks Inc, MA, USA) for pre-processing and subsequent multivariate analysis. The data set was randomly split into training and test cohorts (60:40% ratio) with repeated random sampling (bootstrap), until there was no significant difference (*p* = 0.20) between the two cohorts with respect to all variables. The training set was used for model construction. A specific type of cross-validation was employed—the CV LOO, in which each sample from the training set was evaluated using the model built by all the other samples in the same training set. This was used to check and define important parameters. The remaining samples assigned to the test set were used to evaluate the model performance. For the Artificial Neural Network (ANN) models in the regression mode, all the data were used, and sample selection differed by using the Kennard-Stine method with 60% for training and 40% for testing. Subjects with missing values were omitted. Following the pre-processing stage, all the quantitative variables were normalized. Categorical variables were transformed into binary dummy variables. Next, different subsets of data were labeled to solve the classification prediction problem, namely CCU admission, as positive or negative. This was then formulated as a binary classification problem. For all methods and to avoid over-fitting or selection bias, a leave-one-out cross-validation (VB-CV) was applied to test the model’s ability to predict new data by computing a statistic on the left-out sample. Only subjects with fully curated data were eligible for the prediction analysis.

### 2.4. Multivariate Analysis

Initially, the model was evaluated variable by variable in a univariate fashion, but there was no trend observed. Various state-of-the-art supervised classifiers, suitable for the type and size of the data set, were tested in a multivariate fashion. The algorithms were chosen because of their relative simplicity for interpretation and overall popularity within the data science community and through our own testing of several ML algorithms during model development, focusing on different types of interactions [19]. Algorithms included the non-linear distance models using neural networks (K-Nearest Neighbors (KNN), ANN in Backpropagation Neural Network (BPN) mode, and the models using linear and quadratic discriminant methods (LDA and QDA)). A popular ML model is the ANN; roughly modeled around the way human brain works, it is useful for supervised prediction tasks [20]. Its variation, the BPN, is a type of neural network training that fine-tunes the weights of a neural network based on the error rate obtained from the previous iteration. Proper tuning of the weights allows for reduction of error rates and makes the model reliable, thus increasing its generalizability. This method helps to calculate the gradient of a loss of function with respect to all the weights in the network. A basic prediction technique, such as KNN is employed to verify the complexity of the prediction problem. Linear logistic regression (LR) was used as a benchmark. The conceptually simple approach of LDA and its sibling, QDA, remain among the most effective procedures in the domain of high-dimensional prediction [21,22]. 

### 2.5. Model Performance

To fully evaluate the discriminatory effectiveness of the model, we calculated several performance metrics including accuracy, sensitivity, specificity, F-scores, and the area under the receiver operating characteristic curve (AUC) for the test set. The accuracy represents the total number of samples correctly classified considering true and false negatives. We examined the model performance including all the variables as well as the simplest model with the highest and more robust performance (less features). The sensitivity represented the proportion of positive samples correctly classified. The specificity represented the proportion of negative samples correctly classified and finally, the F-score measured the model performance considering imbalanced data. A Receiver Operating Curve (ROC) was generated as a single metric that is not dependent on the discrimination threshold.

### 2.6. Development of the CCU Risk Calculator

For clinical utility, and to generate real-time predictions, numerous CCU prediction tests at different model probability output thresholds were performed in the validation set, and checked in the derivation set. Because most of the data were classified into one group or another with no intervals, we developed a similar method, using the ANN(BPN) model in calibration mode to turn discrete values into continuous values. A total of five features were selected as threshold, given their significance, and offering the best compromise of computing time required and possible information loss. A certain range of values, minimum and maximum were set for each feature, as below:

Surgical Complexity Score: 2–18;
Albumin levels: 17–49;
Estimated Blood Loss: 100–4000;
Operative Time: 65–445;
Bowel Resection: 0–1;

A software tool (Calibration Tools Hall, UFRN, Natal, Brazil, upon request) by using the ANN(BPN) regression algorithm was developed based on five selected predictor features. The algorithm is a method of fine-tuning the weights of a neural network based on the error rate obtained in the preceding iteration. Proper weight tuning reduces the error rates rendering the model reliable, and increasing its generalizability. Entering values above or below made the Graphical User Interface reset to the closest minimum/maximum value in one or all input variables. All values were numerically entered, and the ‘Estimate Risk’ button was pressed to obtain the estimate in three different formats that complemented each other as a result. The combination of these five features would yield 30 multivariate sets of predictors to evaluate in three different modeling thresholds. These thresholds were influenced by the data distribution and clinical importance of various cut-offs; the entire data set, merging both training and test sets, was then used to report the CCU admission probability percentages. The development of the predictive models and the report of the findings were used in accordance with the EQUATOR Guideline for reporting ML predictive models [23], in support of the STROBE statement for reporting of observational studies [24].

## 3. Results

**Figure 1:**
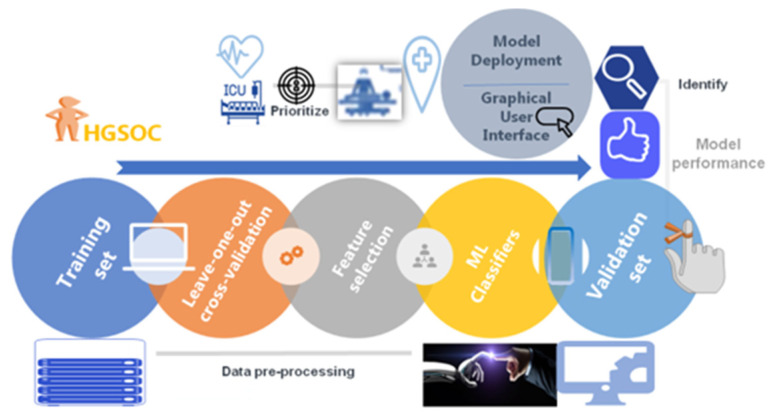
Machine Learning (ML) Model derivation and validation flow from a fully curated source of advanced stage high grade serous ovarian cancer patient and surgical data. The framework comprised data pre-processing with feature selection and model training. The model was evaluated using performance metrics prior to the development of an ML-based nomogram and a web application to help with risk assessment.

**Figure 2:**
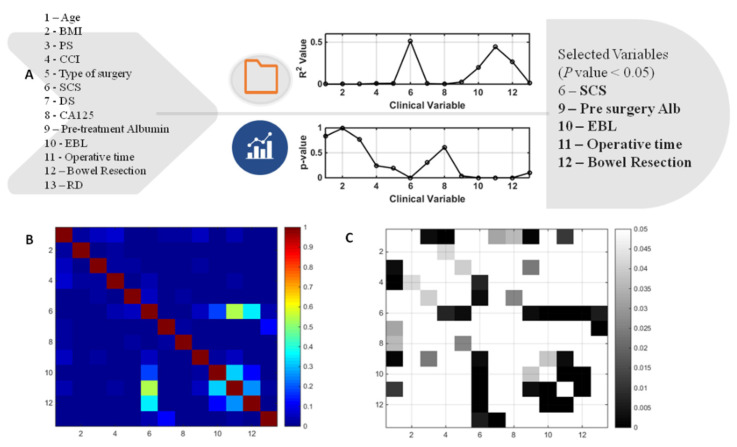
(**A**) Results of multivariate logistic regression leading to feature selection in the development of the predictive Machine Learning model. (**B**) Correlation heatmap representing the values of the determination co-efficient (R2) between the original variables entered for model development. Not surprisingly, the highest correlation was observed between surgical complexity and operative time. (**C**) Correlation heatmap representing the *P* values between the original system variables. In areas where there was no representation (square in any key), there was no significant correlation amongst variables (*p* > 0.05). BMI; body mass index, PS; performance status, CCI; Charlson co-morbidity index, SCS; surgical complexity score, DS;disease score, EBL; estimated blood loss, RD; residual disease, Alb; albumin.

**Figure 3:**
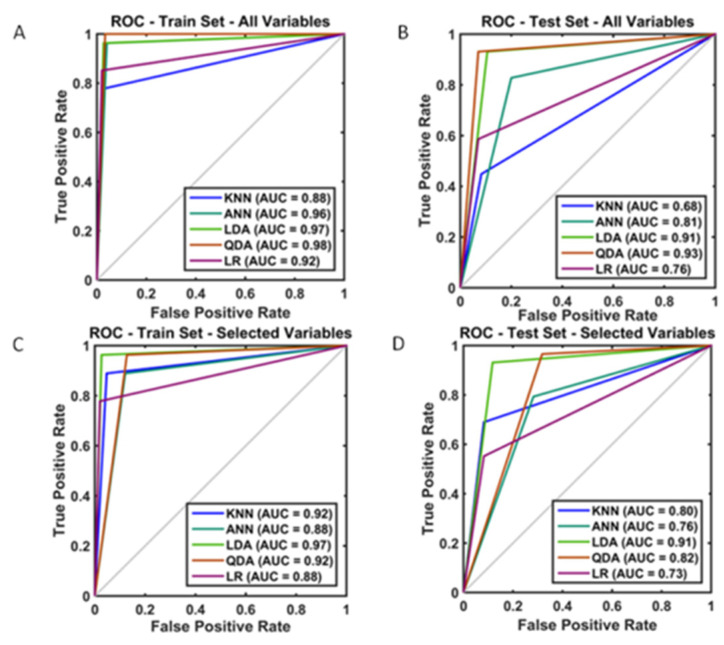
Visual summary of the model performance and evaluation based on the comparison of Receiver Operator Curve (ROC) and Area Under (ROC) (AUROC) for Critical Care Unit admission (**A**) training set; all variables included (**B**) test set; all variables included (**C**) training set; selected variables (**D**) test set; selected variables included.

A general architecture of our proposed methodology is showing in Figure 1. A cohort of 291 patients was eligible for the study. The mean age and BMI of the patients were 64 ± 10.5 yrs, respectively. The mean SCS was 3 ± 2 (2–11). Of these patients, 69/291 (23.7%) and 222/291 (76.3%) underwent PDS and IDS, respectively. Complete and optimal cytoreduction was achieved in 190/291 (65.3%) and 78/291 (26.8%) patients. The details of baseline characteristics and operative factors are displayed in Table 1 to enable the better understanding of this group of patients undergoing cytoreductive surgery and potentially requiring CCU admission. There were 56/291 (19.2%) admissions; of those, 49 admissions were elective whilst seven were unplanned admissions. From the variables selected to predict CCU admission, the following variables were significantly different: pre-treatment albumin, SCS, EBL, OT, and performance of bowel resection with stoma formation (Figure 2). The correlation structure of pre-processed features was demonstrated by a correlation heat map, as well as the most significant variables in Figure 3.

One sample from the training set was omitted due to missing values, totaling 290 patients with complete data, aiming to predict CCU admission. When all the variables were employed, the training accuracy from the derivation set ranged from 0.93 to 0.97. The corresponding internally validated (test) predictive accuracies for the KNN, ANN, LDA, and QDA algorithms were 0.80, 0.82, 0.90 and 0.93, respectively. All but one F-score were convincingly >0.80 (Table 2). Following feature reduction, we observed a slightly worse, yet satisfactory performance with the mean accuracy across classifiers between 0.76 and 0.89 for the CCU admission prediction (Table 2). In that same table, the predictive comparison between ML algorithms and LR suggested a significant outperformance for LDA and KNN over LR. To guide model choice, a visual summary of all the operational algorithms performed can be seen in Figure 3 for both the training and testing partitions. Model performance remained robust between the training and test sets. When all the variables were included, the QDA model performed best in the training set (AUC: 0.98) (Figure 3A). With feature selection, the smallest, highest-performing, and most robust model was the LDA model in the training set (AUC: 0.97) (Figure 3C), yet it performed best in the test set (AUC: 0.91) (Figure 3D). 

### 3.1. Model Deployment

**Figure 4:**
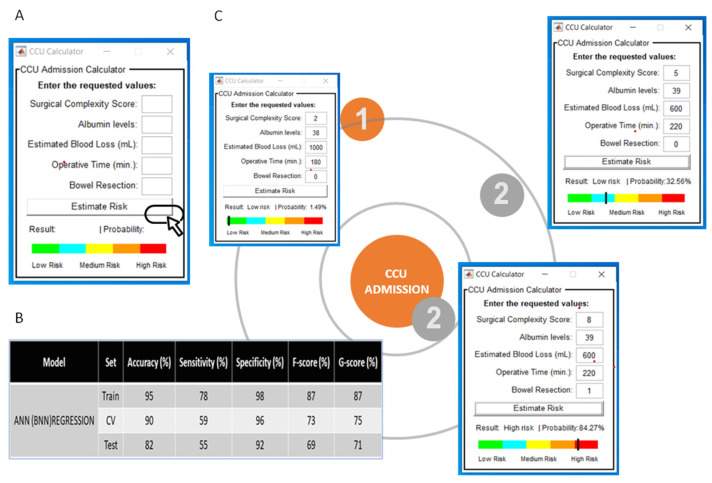
(**A**) Screenshot from the Critical Care Unit (CCU) calculator Graphical User Interface for the prediction of CCU admission. (**B**) Performance of the Artificial Neural Network (ANN(BPN)) algorithm used for the development of the Graphical User Interface based on the regression principles. (**C**) Examples of clinical application. Note that in the same patient, intra-operative decision making for rectosigmoid resection and stoma formation would massively increase the risk for CCU admission.

To assess the clinical utility of our model, a user-friendly Graphical User Iinterface of the CCU admission (the Leeds-Natal) risk score software macro was developed to guide the observers through the analysis. The interface is available upon request. In brief, the interface provided probability results and visualization of the risk position for CCU admission for clinical implementation and easy interpretation by the physicians (Figure 4A). The model performance using the ANN(BPN) regression principle was akin to the ML algorithms used (Figure 4B). We describe the implementation of our method in two HGSOC patients, who underwent cytoreductive surgery (Figure 4C). Patient 1 underwent a standard total abdominal hysterectomy, bilateral salpingo-ophorectomy, and omentectomy following primary chemotherapy. This was a difficult procedure, completed in three hours with an EBL of 1lt. Her probability of getting admitted to CCU was only 1.49%, and she was considered low risk. Patient 2 underwent a standard primary surgical cytoreduction, in addition to lymph node dissection and pelvic peritoneal stripping. The EBL was 600 mL, and the procedure was completed in 220 min. Her risk for CCU admission was 32.56% and she was still considered low risk. If a rectosigmoid resection was anticipated intra-operatively in that same patient, resulting in stoma formation, her SCS would increase from 5 to 8, and her risk for CCU admission would rise to 84.27%. 

### 3.2. Association between CCU Admission and Length of Stay, Post-Operative Complications, and Hospital Re-Admission

Admission to CCU was associated with increased length of stay (*p* = 0.000; OR 8.0, CI 7.7–8.35) and reduced risk for post-operative CD (3a-5) complications, (*p* = 0.001; OR 0.16, CI 0.057–0.45) but no risk for re-admission within 30 days (*p* = 0.63).

## 4. Discussion

In HGSOC, the recent paradigm shift towards achieving complete surgical cytoreduction has certain ramifications to prevent an anticipated increased peri-operative morbidity and mortality [25]. For such a heterogenous disease, the accuracy of any related outcome can be limited, prompting the development of new tools to improve prediction. In this cohort study, we developed and fine-tuned several ML prediction algorithms that accurately predicted CCU admission. Equally, we determined the importance of non-inherent, hence potentially modifiable, prediction features. To our knowledge, this is the first study to quantify patterns of CCU admission following cytoreductive surgery for HGSOC by using ML methods.

Our study allowed for the comparison of several learning algorithms to identify the approach with the most favorable performance. We demonstrated the utility of linear discriminants (LDA, QDA) of clinical characteristics in model performance with maximum accuracy reaching 98% and the lowest boundary of performance being no worse than 68%. The overall model was rendered stable, owing to the quality of the fully curated training data set. Although the study was not powered to the outcome prediction, the data sample was large enough to capture any potential variance. Collinearity was successfully addressed and reduced by feature selection, when required. In addition, we selected supervised ML algorithms such as LDA, QDA, and KNN, which outperformed conventional LR with respect to prediction accuracy indices, which is potentially more clinically relevant than AUC. Such a comparison modeling system has already been shown to test intra-institutional performances [24]. We are familiar with the performance of the KNN algorithm, which is very much reflective of “previous clinical experience”, for the accurate prediction of R0 resection during HGSOC surgery [8]. Where the data classes were unbalanced, the tested methods performed similarly to LR.

Confirming findings from prior empirical research, in addition to the premises of this present work, our study identified those features with the maximal relevance to the CCU admission prediction accuracy. All models placed importance on variables with known predictive implications. All but one was intra-operative parameters. We acknowledge that for planning purposes, it would have been more useful to predict CCU admission based on more pre-operative variables; this was not possible due to the feature selection process. Nevertheless, similar performance metrics were observed when all the variables were included in the model. This highlights the value of the relative importance of each predictor in developing the model. The significant variables may be differentially weighted in the model analysis to reflect estimates of their contribution to relevant similarity. Some discriminants have been previously shown to be predictors of short-term outcomes in EOC patients [13]. Concurrent bowel resection with stoma formation, as part of a frequently extensive cytoreductive surgery, was a critical predictor of CCU admission. This agrees with findings from other studies [26]. It can be explained by the increased risk of severe post-operative complications in patients who undergo rectosigmoid surgery during pelvic exenteration [27]. Advanced stage HGSOC is commonly associated with weight loss, severe malnutrition, and impaired gastrointestinal function, hence low pre-treatment albumin, resulting in a protracted surgical recovery [28]. Primary chemotherapy could potentially impact some of these factors, as shown to decrease intra-operative blood loss, earlier resumption to ambulation, and return of intestinal function [29,30]. The effect of NACT on post-operative admission to CCU remains controversial. Although primary chemotherapy is associated with lower peri-operative morbidity and post-surgical mortality [31], this was not correlated to CCU admission. New systemic treatment algorithms for newly diagnosed advanced EOC could serve as tools to help with clinical decision making and patient stratification on a daily basis [32].

Accurate identification of HGSOC patients at a quantified risk for CCU admission following their surgery is important in gynaecologic oncology. Our findings demonstrated that ML algorithms are able to predict a patient’s risk for CCU admission with excellent discrimination. The main strength of the model was versatility, allowing for case mix when auditing the CCU admission as a proxy indicator of the quality of care. As specificity was high for most models, this would make it attractive in a cancer screening program; if diagnostic prediction is the goal, whereas both sensitivity (true positives) and specificity (true negatives) are important, our highly performed linear discriminant algorithms (LDA and QDA) would primarily apply. Nevertheless, such a tool could be useful in aiding a clinician’s risk assessments in personalized management plans for patients. The development of the first to our knowledge CCU risk calculator—the Leeds-Natal score—provided a fine balance between high model accuracy and model simplicity that makes it easily interpretable to the gynaecologic oncology surgeon. Based on five significant features, it can evaluate which HGSOC subpopulation has the best prediction or which subpopulation is most difficult to predict. In addition, it gives off certain advantages, such as the percentage of those belonging to the “closest to admission” group, which is a ‘true idea’ of the real probability of fitting in the group of women who should be admitted to CCU (Figure 4).

The rate of CCU admission was <20%. The length of CCU stays has been always topical as extensive fluid resuscitation during surgery, poor nutritional status, and older age are associated with a prolonged CCU stay [33]. A breakdown of planned and unplanned admissions reflecting towards objective criteria with respect to valid peri-operative goal-directed haemodynamic therapy protocols is an important topic, and research is currently under way. Herein, although the CCU admission rate increased transiently from 2014 to 2019, the rate of unplanned admissions remained steady (data not shown). In HGSOC, unplanned admissions are associated with reportedly poor survival outcomes [34]. In high-output tertiary centres, interdisciplinary meetings to allocate and prioritize CCU beds prior to the surgical cases may have translated into the observed increased planned and decreased unplanned post-operative CCU admissions.

Prolonged hospitalizations, CCU stays, and hospital re-admissions, have all come under scrutiny due to increasing emphasis on improving palliative care and quality of life for patients near their end of life [35]. Admission to CCU contributes to increased costs for both patients and hospitals [36]. In our post-hoc analysis, CCU admission has been independently associated with increased hospitalization, fewer post-operative complications, but no effect on the risk of re-admission within 30 days, in contrast to other studies [34]. If extensive surgery is associated with morbidity-related infections and wound complications, it may be prudent to provide nutrition support ensuring the peri-operative period in these women [37]. In that same report, CCU admission increased the odds of severe surgical infections, compared to routine ward admission. For such an inherently high-risk population, this information is critical when counseling women about peri-operative risks ahead of their surgery, and potential adverse impacts in their overall survival.

This was a single-centre study with a limited rate of heterogeneity in the study population, which may differ from other tertiary unit settings. The retrospective nature of the study was the main limitation, despite a proprietary, prospectively maintained database. A very slight discordance of model performance between the training and test sets may not be generalized to other data sources. Nevertheless, ML retains the strength of the structural model used for the prediction, even when applied to other populations with different prediction features. Although model robustness was strongly supported by internal validation, further external validation in an independent cohort may be required to confirm the ability of the models as guidance tools.

## 5. Conclusions

In high-output centres, CCU admission is a measurable outcome that can be used as a benchmark of surgical care. We developed ML models that predicted CCU admission following cytoreductive surgery for HGSOC with an accuracy as high as 98%. Equally, we refined risk-adjusted predictors of CCU admission using pertinent pre-operative and intra-operative variables. Most importantly, we demonstrated clinical feasibility by the development of a Graphical User-friendly Interface to quantify patterns of CCU admission. Predictive ML algorithms may facilitate quality improvement of modern care by improving the CCU admission prediction accuracy.

## Figures and Tables

**Table 1:** Pre-operative, intra-operative and post-operative parameters of the study cohort.

Variables (*n* = 291)	Levels	Frequency	Percentage
Age, year, SD (range)	N/A	64.2 ± 10.5 (41–90)	N/A
BMI, mean, SD (range)	N/A	27 ± 5.8 (18.5–58)	N/A
Pre-Treatment CA125, mean, SD (min-max)	N/A	1777 ± 3125 (12–28,000) U	N/A
Pre-Treatment Albumin, mean, SD (min-max)	N/A	38.3 ± 3.8 (17–49) U	N/A
Surgical Complexity Score (SCS)	Low (1–3)	166	57%
	Moderate (4–7)	108	37.1%
	High (8–12)	17	5.9%
Disease score (DS)	Pelvis	17	5.8%
	Lower abdomen	220	75.6%
	Upper abdomen	54	18.6%
Residual Disease (RD)	R0	190	65.3%
	R1 (<1 cm)	78	26.8%
	R2 (≥1 cm)	23	7.9%
CCU admission	Yes	56	19.2%
	No	235	80.8%
Bowel resection with stoma	Yes	21	7.2%
	No	270	92.8%
ECOG Performance Status (PS)	0	127	43.6%
	1	119	40.9%
	2	38	13.1%
	3	7	2.4%
Charlson Co-morbidity Index (CCI)	Low (0–2)	146	50.2%
	High (≥3)	145	49.8%
Timing of cytoreduction	PDS IDS	69 222	23.7% 76.3%
Operation time, mean SD (min-max)	N/A	182 ± 75 (65–480) min	N/A
Length of stay, mean, SD (min-max)	N/A	9 ± 13 (3–172) days	N/A
Clavien-Dindo complications (3a–5)	Yes	16	5.5%
Admission within 30 days	Yes	Data	Data

ECOG; Eastern Co-operative Oncology Group; PDS; primary debulking surgery, IDS; interval debulking surgery.

**Table 2:** Performance metrics of the Machine Learning models and comparisons with conventional Logistic Regression for the prediction of Critical Care Unit admission following cytoreductive surgery for advanced high grade serous ovarian cancer.

Predictors	Model	Set	Accuracy	Sensitivity	Specificity	F-Score
All variables (*n* = 13)	KNN (K = 4)	Train	0.94	0.78	0.97	0.86
		CV LOO	0.94	0.78	0.97	0.86
		Test	0.80	0.45	0.92	0.60
	ANN	Train	0.97	0.96	0.97	0.96
		CV LOO	0.88	0.85	0.88	0.86
		Test	0.82	0.86	0.81	0.83
	LDA	Train	0.97	0.96	0.97	0.96
		Test	0.90	0.93	0.89	0.91
	QDA	Train	0.97	1.00	0.97	0.98
		Test	0.93	0.93	0.93	0.93
	LR	Train	0.96	0.85	0.98	0.91
		Test	0.84	0.59	0.93	0.72
Selected * Variables (*p* < 0.05)	KNN (K = 6)	Train	0.94	0.89	0.95	0.92
		CV LOO	0.94	0.89	0.95	0.92
		Test	0.86	0.69	0.92	0.79
	ANN	Train	0.90	0.89	0.90	0.99
		CV LOO	0.89	0.89	0.89	0.89
		Test	0.76	0.79	0.74	0.76
	LDA	Train	0.97	0.96	0.97	0.96
		Test	0.89	0.93	0.88	0.90
	QDA	Train	0.89	0.96	0.87	0.91
		Test	0.75	0.97	0.68	0.80
	LR	Train	0.95	0.78	0.98	0.87
		Test	0.82	0.55	0.92	0.69

* Surgical complexity score; pre-surgery albumin; blood loss; operative time; bowel resection with stoma. KNN; k-Nearest Neighbors, ANN; Artificial Neural Network, CV-LOO; Leave-one-out-cross-validation; LDA; Linear Discriminant Analysis, QDA; Quadratic Discriminant Analysis, LR; Logistic Regression.

## Data Availability

The data presented in this study are available on request from the corresponding author.
